# Scorpionism in the Amazon: Evidence, Challenges and Actions for Controlling a Neglected Disease

**DOI:** 10.1590/0037-8682-0497-2025

**Published:** 2026-06-22

**Authors:** Jacqueline Sachett, Fan Hui Wen, Elisangela Aparecida da Silva, Alexandre Vilhena da Silva, Zehev Benzaken, Alessandro Júnio Campelo Feitosa, Yasmim da Silva Mendes, Handerson da Silva Pereira, Pedro Ferreira Bisneto, Maria Cristina Martins de Oliveira, Felipe Murta, Marco Aurélio Sartim, Wuelton Monteiro

**Affiliations:** 1 Universidade do Estado do Amazonas, Escola Superior de Ciências da Saúde, Manaus, AM, Brasil.; 2 Fundação de Medicina Tropical Dr. Heitor Vieira Dourado, Departamento de Ensino e Investigação, Manaus, AM, Brasil.; 3 Instituto Butantan, Centro Bioindustrial, São Paulo, SP, Brasil.; 4 Universidade Federal do Amazonas, Faculdade de Ciências Farmacêuticas, Manaus, AM, Brasil.; 5 Universidade Federal do Amazonas, Instituto de Ciências Biológicas, Manaus, AM, Brasil.; 6 Duke University, Duke Global Health Institute, Durham, NC, USA.

**Keywords:** Scorpion stings, Tityus, Epidemiology, Amazonia, Research agenda

## Abstract

Scorpion sting envenomations impose an increasing burden in the Amazon and result in a considerable impact on public health. The genus *Tityus* accounts for nearly all clinically relevant cases, with *T. metuendus*, *T. silvestris* and *T. obscurus* being responsible for most of envenomations in the region. In the state of Amazonas, Manaus and Apuí account for most of cases reported, with an evident expansion to other regions, including the Solimões River region and the Upper Negro River region, where most of the exposed population is found in Indigenous and riverine communities. The increasing burden and high severity rates of scorpion stings in the Amazon may result from the capacity of adaptation of the endemic species to the environmental changes and limitations in providing adequate healthcare to patients. The clinical profile of patients stung by different species has similar local and systemic manifestations. In Manaus, *T. metuendus* is the species that is most commonly responsible for severe cases observed in children, due to them presenting circulatory collapse and acute respiratory distress, while *T. apiacas* and *T. silvestris* present systemic neurological manifestations, characterized by generalized muscle spasms described as the sensation of receiving an electric shock, apparently not reversed by the available antivenom. Scorpionism in the Amazon combines the biological complexity and variability of *Tityus* species and their venoms, social and environmental determinants and healthcare challenges. These features require solutions of interinstitutional, interdisciplinary networks that are capable of integrating biology, venomics, clinical medicine, public health, environmental sciences and the social sciences.

## TEMPORAL EVOLUTION AND CURRENT SITUATION OF SCORPION ENVENOMATIONS IN THE STATE OF AMAZONAS

Scorpionism is recognized as an increasingly important public health problem in many tropical countries, including Brazil. It is estimated that more than 1.2 million stings occur annually, with ~3,250 deaths, especially in tropical and subtropical regions that are characterized by unplanned urbanization, inadequate sanitation and social inequities^1^ ^-^ ^5^.

In Brazil, the genus *Tityus* accounts for nearly all clinically relevant cases, with *Tityus serrulatus* being historically linked to severe envenomation in the southeastern region^6^. In 1988, scorpion stings and spider bites were included in the National Program for Epidemiological Surveillance two years after the creation of the National Snakebite Program, renamed as the National Program for the Control of Accidents by Venomous Animals. The first data available at national level indicated the occurrence of 7,500 cases in 1988-1989, and an incidence rate of 2.53 cases/100,000 inhabitants. Less than 1% came from the northern region^7^.

**FIGURE 1:**
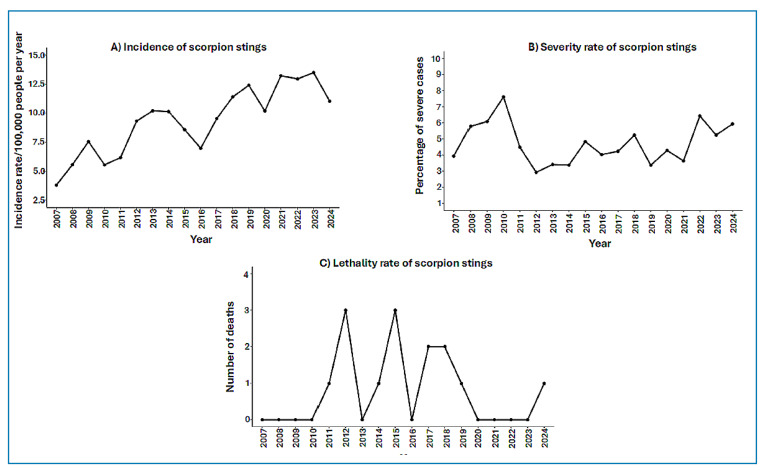
Historical time series of scorpion envenomations in the state of Amazonas, Brazil (2007-2024)^9^.

Over recent decades, the geographic distribution of medically important scorpions has expanded, accompanied by a marked rise in case notifications. This trend reflects both the ecological adaptability of scorpions and structural limitations within the health system to provide equitable care^8^. More reliable data has been available since 2007, from the National Notifiable Diseases Information System (SINAN)^9^, which show a significant increase in the number of notifications in the state of Amazonas, coincident with the evolution of cases at the national level^10^ (Figure 1).

In 2007, fewer than 150 cases were recorded in Amazonas, which were concentrated mainly in Manaus and some neighboring municipalities. From 2010 onward, an upward curve is evident, with annual averages exceeding 300 cases and peaks surpassing 800 notifications in more recent years. Studies carried out using data collected from SINAN between 2007 and 2014 indicated an incidence rate of 7.6 per 100,000 inhabitants/year^11^ and 8.14 per 100,000 inhabitants^12^ in Amazonas, lower than the Brazilian average of 32 cases/100,000 inhabitants. In Brazil, the northern region-comprising states within the Amazon ecosystem-displays one of the lowest incidence rates of scorpionism in the country; however, it concurrently presents the highest lethality rates, despite the comparatively low overall frequency of cases^10^.

Spatial distribution showed a large swatch in the southern region of the state, where scorpion stings were predicted to have incidence rates of > 50 cases per 100,000 inhabitants/year, with the highest mean incidence reported in Apuí with 183.8 cases per 100,000 inhabitants/year. Manaus and its surrounding areas also were also higher than 50 cases per 100,000 inhabitants/year^11^. 

**FIGURE 2:**
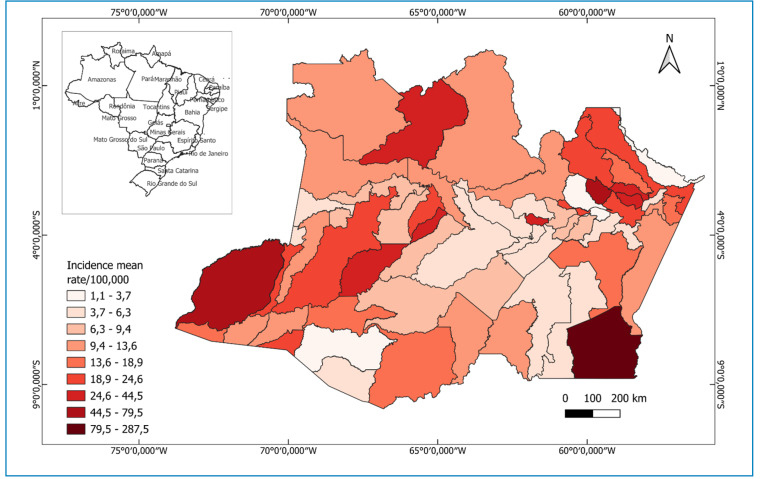
Distribution of scorpionism incidence rates (cases/100,000) in Amazonas state from 2021-2024.

Currently, cases are concentrated in Apuí and Manaus (60-70% of annual notifications). with an increasing number of cases in medium-sized locations such as Itacoatiara, Manacapuru, Parintins and Tefé, as well as emerging notifications in remote communities along the Solimões River and in the Upper Negro River region. This pattern may reflect both the increased presence of scorpions in poor and recently urbanized settings, in which the rapid and unplanned urbanization of originally forested areas, without adequate infrastructure and services, create favorable conditions for the proliferation of scorpions. In addition, improved capacity for detection and reporting in locations that were previously under-reported has occurred, which is observed in the SINAN maps (Figure 2 **).** These show a transition from an initially Manaus-centered scenario to a statewide context, with the majority of the 62 municipalities reporting cases in 2023^13^. Probable under-notification persists in indigenous and rural areas since access to healthcare is limited.

## DISTRIBUTION OF SCORPIONS AND BURDENOF SCORPIONISM IN THE AMAZON REGION

### Scorpions of medical importance in the Amazon region

**FIGURE 3:**
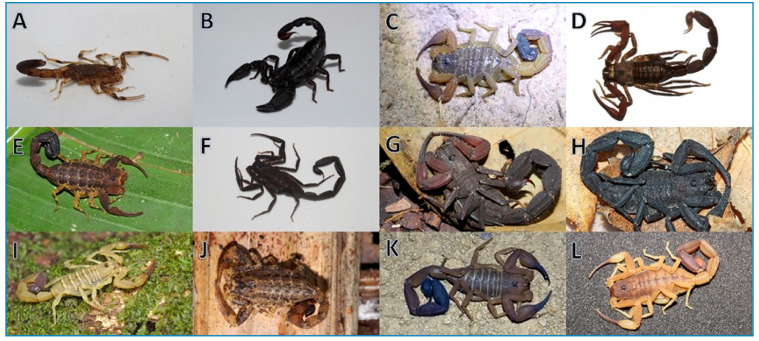
Scorpions involved in envenomations in the Brazilian Amazon. **(A)** *Ananteris dekeyseri*; **(B)** *Brotheas amazonicus*; **(C)** *Rhopalurus laticauda*; **(D)** *Tityus apiacas*; **(E)** *Tityus bastosi*; **(F)** *Tityus dinizi*; **(G)** *Tityus metuendus*; **(H)** *Tityus obscurus*; **(I)** *Tityus raquelae*; **(J)** *Tityus silvestris*; **(K)** *Tityus strandi*; and **(L)** *Tityus sylviae*.

The Amazon harbors a high diversity of Neotropical scorpions, encompassing four families: Buthidae, Chactidae, Ischnuridae and Troglotayosicidae^14^. Only members of Buthidae and Chactidae have been implicated in human envenomations (Figure 3). *Brotheas amazonicus* (Chactidae) and *Ananteris dekeyseri* (Buthidae) have caused only mild local symptoms, while *Rhopalurus laticauda* (Buthidae) is suspected of causing envenomation in the far north, although no confirmed cases have been reported^15^.

The genus *Tityus* (Buthidae) comprises the most medically relevant species, including *T. obscurus*, *T. metuendus*, *T. silvestris*, *T. apiacas* and *T. strandi* ^8^ ^,^ ^16^ ^-^ ^18^. Their distribution is heterogeneous, spanning terra-firme rainforests, igapós and várzea floodplains, forest edges and peri-urban environments, where urban expansion favors opportunistic species^14^ ^,^ ^19^. *T. obscurus*, *T. metuendus* and *T. silvestris* account for most severe cases^16^ ^,^ ^20^, occurring respectively in the eastern, central/western and in the entire Amazon^21^ ^,^ ^22^.

These species inhabit primary forests but also persist in secondary fragments and anthropic settings associated with deforestation, fire and urbanization, which reconfigure communities and trophic interactions^8^ ^,^ ^14^ ^,^ ^18^ ^,^ ^23^. Amazonian scorpions are nocturnal and cryptic, occupying leaf litter, bark, logs, bromeliads and crevices; seasonal flooding modulates activity and increases encounters with humans^12^ ^,^ ^21^.

### Influence of climate changes on public health risks for scorpion stings

The increasing incidence rate of scorpionism and expanding geographical distribution of scorpions have been observed in different countries, such as Argentina, Mexico and Iran^24^ ^-^ ^27^. In Brazil, studies have focused different *Tityus* species and the leading challenges for public health^25^ ^,^ ^28^ ^-^ ^31^. 

The increasing burden and high severity rates of scorpion stings in the Amazon may result from the capacity of adaptation of the endemic species to the environmental changes and limitations in providing adequate healthcare to patients. Theoretical models have been developed to predict the burden of scorpionism caused by Amazonian *Tityus* species under different climate scenarios. *T. silvestris* and *T. metuendus* demonstrated greater resilience to climate change, with predictions indicating stability and even expansion of their geographical distribution^32^.

Another study hypothesized a reduction in suitable habitats for *T. serrulatus*, while for *T. metuendus* and *T. silvestris*, models indicated an increased abundance of these species in response to bioclimatic factors, such as temperature and precipitation shifts, and socioeconomic aspects of Amazonian cities, such as urban infrastructure, which facilitates the presence of scorpions in areas with poor sanitation and waste management^32^. 

## ADVANCING THE SCIENTIFIC KNOWLEDGE ON DIAGNOSIS, TREATMENT AND CONTROL OF SCORPION STING ENVENOMATIONS IN THE AMAZON

### Venom and pathophysiology

Scorpion venom comprises a complex mixture that is dominated by neurotoxins that target voltage-gated sodium channels (Nav)-both α- and β-toxins, typically 60-76 amino acids with multiple disulfide bonds-together with potassium channel toxins (KTx) peptides (blockers of voltage-gated potassium channels (Kv); several subfamilies), enzymes (e.g., hyaluronidases), low-abundance phospholipases, antimicrobial peptides, and accessory components (binding proteins and regulatory factors). One of the most striking differences between Amazonian scorpion venoms and those from other regions is their biochemical diversity and regional variability, even within the same species. Proteo/transcriptomic (“venomic”) analyses of *Tityus obscurus* and *T. metuendus* indicate a high investment in ion-channel-active toxins, with compositional profiles that differ from *T. serrulatus* ^33^ ^,^ ^34^. Marked heterogeneity is evident across species, populations, ontogenetic stages (immature vs. adult), sexes, diet and environmental settings (e.g., Amazonian terra-firme gradients vs. várzea gradients). This variation aligns with the distinct clinical profiles reported for Amazonian species and helps explain suboptimal neutralization by antivenom produced against *T. serrulatus* ^19^ ^,^ ^35^ ^,^ ^36^.

Comparative analyses of venoms from Amazonian *Tityus obscurus* and *T. metuendus*, and the endemic *T. serrulatus* from other regions, reveal clear biochemical and evolutionary differences, highlighting the largely unexplored diversity of Amazonian scorpions. *T. obscurus* venom is particularly distinctive, since it is rich in non-disulfide bridged peptides, including novel fragments related to hypotensins, KTx, and allergen-like proteins-components that are absent in *T. serrulatus*. Phospholipase A₂ is confirmed only in *T. obscurus*, while phospholipase C and D transcripts are present in both species but remain unverified at the protein level. *Tityus obscurus* venom spans a broad molecular range: high-mass enzymes include hyaluronidases, metalloproteinases and cysteine proteinases; mid-mass venom proteins/toxins proteins feature Cysteine-Rich Secretory Proteins (CRISPs) and serine proteases; low-mass components include proteinase inhibitors. Neurotoxins such as sodium channels toxins (NaTx) and KTx expand its venom arsenal. *T. metuendus* additionally contains bradykinin-potentiating peptides. In contrast, *T. serrulatus* venom is dominated by low-mass neurotoxins responsible for its high lethality, alongside higher-mass enzymes like metalloproteinases, serine proteinases, phospholipases and hyaluronidases. Unique components absent in Amazonian species include amylase, phosphodiesterase, chitinase and diverse low-mass peptides such as scorpine-like peptides^37^ ^-^ ^40^.

Functionally, the principal drivers of toxicity are peptides that modulate Nav and Kv channels in sensory, autonomic and cardiac fibers: α-toxins delay Nav inactivation (prolonging Na⁺ current) and thereby increase neuronal excitability; β-toxins shift the voltage sensor to favor channel opening at more negative potentials, triggering spontaneous discharges; and KTx peptides block Kv currents, diminishing repolarization, prolonging action potentials and compounding hyperexcitability. The distribution of Nav subtypes-Nav1.7-Nav1.9 in nociceptors and Nav1.4/1.5/1.6 in skeletal muscle, the heart and neurons-maps well onto the algoneurologic phenotype and autonomic manifestations observed clinically^41^ ^-^ ^43^. 

Excessive peripheral and central autonomic drive produces a mixed, often oscillating autonomic storm, with catecholaminergic signs (tachycardia/hypertension, diaphoresis, mydriasis) and parasympathetic features (sialorrhea, vomiting, abdominal cramps). In children, a smaller volume of distribution and higher venom-to-body-mass ratio exacerbate autonomic instability^19^ ^,^ ^36^ ^,^ ^41^ ^,^ ^44^. Catecholamine surges and autonomic dysfunction may precipitate tachy-/bradyarrhythmias and hyper-/hypotension. Two mechanisms of pulmonary edema are described: cardiogenic (hypertensive surge/afterload with transient myocardial dysfunction) and non-cardiogenic (increased permeability mediated by inflammation/neuropeptides). Shock can occur in severe cases^41^ ^,^ ^45^ ^,^ ^46^.

 In addition to local pain and paresthesia, Amazonian case series report atypical neurologic phenotypes-diffuse “electric-shock” sensations, generalized paresthesia, ataxia and involuntary movements-most frequently associated with stings by *T. obscurus*, *T. silvestris* and *T. apiacas*. A unifying hypothesis invokes a toxin repertoire with distinctive actions on central and peripheral Nav, together with KTx and possible Ca²⁺-channel-modulating peptides^43^. Catecholamine excess may coexist with transient hyperglycemia, leukocytosis and electrolyte disturbances. Prominent gastrointestinal features-intense nausea/vomiting, sialorrhea, abdominal pain-reflect cholinergic/peptidergic hyperactivity. Experimental and clinical data also implicate cytokines (e.g., Interleukin 6, Tumoral Necrosis Factor-α) and neuropeptides in sustaining systemic manifestations^41^ ^,^ ^47^.

### Clinical profile, risk groups and complications of scorpionism in the Amazon

In the Amazon basin, human populations living in close contact with the forest, such as Indigenous people and rural workers, are the most susceptible to scorpion stings. However, climate change and greater human occupation in rural areas have contributed to the dispersion of scorpions to urban areas, where stings have been recorded in ever higher numbers^16^.

In a series of 151 confirmed scorpion stings recorded in Manaus and the surrounding region, and the municipality of Apuí (southern Amazonas), the predominant species were *T. metuendus* (68.2%), *T. silvestris* (14.6%), *T.raquelae* (7.9%) and *T. apiacas* (4.6%)^16^.

*T. metuendus* and *T. silvestris* are known to be responsible for envenomations in the state of Amazonas. Although *T. metuendus* inhabits forest environments, sheltering on trunks, bark and fallen branches, there have been reports of domestic invasions in the metropolitan area of Manaus, with stings occurring in the intra- and peri-domiciliary settings. *Tityus silvestris* has also been involved in envenomations in urban environments. *T. raquelae* envenomation was first described by our group in the adjacencies of Manaus, and in the municipality of Tefé, 600 km from the capital of Amazonas, whereas *T. apiacas* was identified as the cause of envenomations in Apuí^16^.

Epidemiological characteristics showed no differences among patients stung by different species. Males were affected in 53.6% of cases, mostly in the age range of 40 to 49 years (22.5%). Labor activities were involved in 12.6% of cases. Patients are usually bitten on the feet (49.0%) or hands (31.8%). The clinical profile of patients stung by different species was also similar in terms of local and systemic manifestations, systematized as follows:

- Local manifestations: pain (84.1%), paresthesia (34.4%), slight edema (25.8%), hyperemia (21.9%), sting mark (19.2%) and local sweating (3.3%), piloerection (2.0%) and local spasms (1.3%)^16^.
- Systemic manifestations: nausea/vomiting (16.6%); myoclonia (8.6%); lethargy (6.0%), tachycardia and tachypnea (5.3%); cephalea, agitation, sialorrhea and sweating (4.6%), dyspnea (3.3%); abdominal pain (2.6%); blurred vision, diarrhea and hypotension (2.0%); respiratory distress and tremors (1.3%); and convulsions (0.7%)^16^.

Most of the cases were considered to be of Class I severity (80.8%), while Class II and Class III were 15.9% and 3.3%, respectively. *Tityus metuendus* is the species that is most often responsible for severe cases in Manaus, and children are the most susceptible to the occurrence of circulatory collapse and acute respiratory distress^11^, similarly to the envenomations attributed to *T. serrulatus* ^13^ ^,^ ^48^ ^,^ ^49^. 

It is interesting to highlight that patients stung by *T. apiacas* present systemic manifestations, expressed as myoclonia, described as unusual, involuntary and generalized tingling and numbness, reported as an electric shock sensation, affecting mainly the trunk and legs. The same clinical spectrum was described by our group in another four confirmed cases in Apuí^45^. One unusual, confirmed case of a *T. silvestris* sting with severe envenomation was described in an adult patient with generalized muscle spasms, tachycardia and hypertension that evolved with dyspnea, requiring intensive care for monitoring of vital signs and oxygen therapy^50^. These manifestations are similar to those described for envenomation by *T. obscurus* ^36^ ^,^ ^46^ and also reported in envenomations caused by *T. strandi* ^51^ in the state of Pará. In such cases, symptoms lasted for up to 24 hours after the sting. This clinical syndrome has not been reported in envenomations by *T. serrulatus* or *T. stigmurus*, found in other regions of the country^48^ ^,^ ^52^ ^,^ ^53^. Nevertheless, severity criteria of scorpion envenomations in Brazil are based only on the sympathetic signs and symptoms, and do not take into account the possible cerebellar disfunction and effects on skeletal muscle caused by the Amazonian scorpion species. There is no apparent efficacy of antivenom treatment in reducing the intensity and duration of the neurological manifestations^54^. The production of *Tityus* antivenom in Brazil involves the immunization of horses with antigens prepared from *T. serrulatus* venom only, the same occurring for the polyvalent *Loxosceles*, *Phoneutria* and *Tityus* antivenom, in which the anti-scorpion antibodies are generated from the *same T. serrulatus* antigen. Therefore, it is fundamental to perform studies to assess the potential differences in toxin antigenic cross-reactivity between Amazonian species and the current antivenom, as well to establish the ideal therapeutical approach for these scorpion stings^55^. 

## PRIORITY RESEARCH AGENDA FOR SCORPIONISM FOR THE COMING YEARS IN THE AMAZON REGION

Scorpionism in the Amazon combines high biological complexity (diversity of *Tityus* species and venom variability), social and environmental determinants (peri-urban expansion, inadequate sanitation, long travel distances), and healthcare challenges (access to antivenom, logistics and clinical monitoring). These features call for solutions that involve interinstitutional, interdisciplinary networks that are capable of integrating biology, venomics, clinical medicine, public health, environmental sciences and the social sciences^21^ ^,^ ^56^.

Over the past decade, advances in scientific knowledge on scorpionism in Amazonas have been driven by the Tropical Medicine Program at the Amazonas State University (UEA) and the Dr. Heitor Vieira Dourado Tropical Medicine Foundation (FMT-HVD). This program established a line of research on envenomation by venomous animals that expanded beyond snakebites to include scorpion stings. Although the number of scorpionism cases in the Amazon is lower than that of snakebite cases, the topic has already been the focus of master’s theses, doctoral dissertations and postdoctoral projects.

Most recently, a research-linked scorpion colony was initiated at FMT-HVD to support taxonomic identification of Amazonian scorpions, to advance understanding of their biology and behavior and to build a multi-species venom bank. The colony currently includes *Tityus metuendus*, *T. dinizi*, *T. silvestris*, *T. sylviae*, *Ananteris pydanieli*, *Ananteris dekeyseri* and *Brotheas amazonicus*. Therefore, a priority research agenda has been elaborated, covering several themes:

### Estimating the burden of scorpion stings in the Amazon region

1. To estimate the underreporting of the burden of scorpion stings in the Indigenous and riverine populations, through population- and hospital-based field studies in remote areas.
2. To estimate the costs associated with scorpion-sting envenomations in the health system and from a more general societal perspective.

### Agents of scorpion stings in the Brazilian Amazon

1. To carry out field works to describe the composition of the scorpion fauna in different settings, as well as behavioral patterns (diet, reproduction and activities), and ecological alterations that affect the distribution of scorpion populations.
2. To establish phylogeny patterns for identifying risk factors for envenomations.
3. To enable the Amazonian municipalities and states to implement surveillance programs for the control of scorpion species that pose risks to human health, including exotic species (e.g., *T. serrulatus*) that may be highly adapted to the anthropic environment in the Amazon region.
4. To identify urban and rural areas in which environmental factors favor the proliferation of scorpions, and to develop surveillance campaigns in these locations.

### Clinical aspects of scorpion stings in the Amazon region

1. To describe clinical characteristics and complication rates associated with envenomations caused by different scorpion species in the Amazon.
2. To detail the clinical presentation in children and risk factors for the severity of envenomations, and scorpion species correlated with systemic manifestations.
3. To identify the effectiveness of health facilities located in the municipalities of the interior in order to manage scorpion envenomations and their possible complications.

### Venom research: biochemistry and pathophysiology

1. To study the chemical composition of all medically relevant Amazonian scorpion venoms, including the use of “omics” technologies.
2. To define the predominant pathophysiological mechanisms and modes of action of venoms of different populations of scorpions in the Amazon region, considering the possibility of intra-species variations.
3. To verify the association between the chemical composition of all medically relevant Amazonian scorpion venoms and their clinical presentations.

### Therapy issues and antivenom spectrum efficacy

1. To carry out preclinical assessments of existing antivenoms against the venoms of scorpion species in the state of Amazonas.
2. To perform multicenter studies aimed at standardizing clinical protocols for assessing antivenom efficacy and defining objective criteria for recommending antivenom administration and dosage.
3. To undertake phase IV studies for adverse reactions under antivenom pharmacovigilance.

### Network of scorpion envenomation assistance and professional training in the Amazon

1. To organize programs for systematic training for all health professionals, including nurses who are critical in the initial management of the patients, with special emphasis on criteria for antivenom administration.
2. To systematically update all relevant diagnosis and treatment guidelines.
3. To encourage the use of information and communication technologies and diverse electronic media in training programs and distance learning.

## FINAL REMARKS

Over the past two decades, scorpionism in Amazonas, long regarded as sporadic, has consolidated itself as an emerging public-health problem and continues to be less prevalent than in other Brazilian regions. A progressive rise in cases is evident, with a spread into previously non-endemic municipalities and a shift in the profile of the affected population. The region’s environmental, social and cultural specificities confer distinct epidemiological patterns. The Amazon biome harbors high scorpion diversity, including species with unique toxicological profiles, which may shape clinical presentations and therapeutic responses.

Evidence indicates that scorpion stings increase under conditions of unplanned urbanization, precarious housing and limited preventive education, a pattern repeatedly observed in high-incidence regions of Brazil (north, northeast, and southeast). To overcome these challenges, a coordinated strategy is required: (i) health-education and community campaigns tailored to local languages and contexts; (ii) equitable distribution of antivenom, including pre-positioning in remote areas; (iii) integration of epidemiologic and environmental surveillance (One Health approaches); (iv) capacity building and evidence-based clinical protocols across levels of care; and (v) community engagement through citizen science and participatory approaches. When supported by qualitative and quantitative research, these actions can strengthen Brazil’s response to scorpionism and reduce the obstacles that impede timely treatment and control.

Building an Amazon-focused agenda for scorpionism demands sustained funding, close articulation among research institutions, health services and local communities, and respect for sociocultural and environmental specificities. Addressing key gaps-epidemiologic, ecological, clinical and operational-will help reduce disease burden, promote health equity and reinforce scientific sovereignty in the Amazon.

## Data Availability

Research data is available in the body of the article.
